# Circulating acetylated polyamines correlate with Covid-19 severity in cancer patients

**DOI:** 10.18632/aging.203525

**Published:** 2021-09-13

**Authors:** Mélanie Bourgin, Lisa Derosa, Carolina Alves Costa Silva, Anne-Gaëlle Goubet, Agathe Dubuisson, François-Xavier Danlos, Claudia Grajeda-Iglesias, Luigi Cerbone, Arthur Geraud, Ariane Laparra, Fanny Aprahamian, Nitharsshini Nirmalathasan, Frank Madeo, Laurence Zitvogel, Guido Kroemer, Sylvère Durand

**Affiliations:** 1 Gustave Roussy Comprehensive Cancer Institute, Villejuif 94805, France; 2 Centre de Recherche des Cordeliers, Equipe Labellisée par la Ligue Contre le Cancer, Université de Paris, Sorbonne Université, Inserm U1138, Institut Universitaire de France, Paris 75006, France; 3 Metabolomics and Cell Biology Platforms, Gustave Roussy Cancer Center, Université Paris Saclay, Villejuif 94805, France; 4 Institute of Molecular Biosciences, NAWI Graz, University of Graz, Graz 8010, Austria; 5 BioTechMed-Graz, Graz 8010, Austria; 6 Field of Excellence BioHealth, University of Graz, Graz 8010, Austria; 7 Inserm U1015, Villejuif 94805, France; 8 Center of Clinical Investigations in Biotherapies of Cancer (Biotheris), Villejuif 94805, France; 9 Faculty of Medicine, Université Paris Saclay, Le Kremlin-Bicêtre 94270, France; 10 Pôle De Biologie, Hôpital Européen Georges Pompidou, AP-HP, Paris 75015, France; 11 Department of Drug Development (DITEP), Gustave Roussy, Villejuif 94805, France; 12 Cancer Medicine Department, Gustave Roussy, Villejuif 94805, France; 13 Inserm U981, Villejuif 94805, France

**Keywords:** COVID-19, cancer, polyamines, microbiota, metabolomics

## Abstract

Cancer patients are particularly susceptible to the development of severe Covid-19, prompting us to investigate the serum metabolome of 204 cancer patients enrolled in the ONCOVID trial. We previously described that the immunosuppressive tryptophan/kynurenine metabolite anthranilic acid correlates with poor prognosis in non-cancer patients. In cancer patients, we observed an elevation of anthranilic acid at baseline (without Covid-19 diagnosis) and no further increase with mild or severe Covid-19. We found that, in cancer patients, Covid-19 severity was associated with the depletion of two bacterial metabolites, indole-3-proprionate and 3-phenylproprionate, that both positively correlated with the levels of several inflammatory cytokines. Most importantly, we observed that the levels of acetylated polyamines (in particular N_1_-acetylspermidine, N_1_,N_8_-diacetylspermidine and N_1_,N_12_-diacetylspermine), alone or in aggregate, were elevated in severe Covid-19 cancer patients requiring hospitalization as compared to uninfected cancer patients or cancer patients with mild Covid-19. N_1_-acetylspermidine and N_1_,N_8_-diacetylspermidine were also increased in patients exhibiting prolonged viral shedding (>40 days). An abundant literature indicates that such acetylated polyamines increase in the serum from patients with cancer, cardiovascular disease or neurodegeneration, associated with poor prognosis. Our present work supports the contention that acetylated polyamines are associated with severe Covid-19, both in the general population and in patients with malignant disease. Severe Covid-19 is characterized by a specific metabolomic signature suggestive of the overactivation of spermine/spermidine N_1_-acetyl transferase-1 (SAT1), which catalyzes the first step of polyamine catabolism.

## INTRODUCTION

Coronavirus disease-19 (Covid-19) has challenged and transiently overwhelmed the health care system of all Western countries. Infection by severe acute respiratory syndrome coronavirus-2 (SARS-CoV-2), the causative agent of Covid-19 is usually asymptomatic or pauci-symptomatic in healthy individuals [1, 2]. However, old age and age-related diseases are risk factors that predispose to a more severe disease course requiring hospitalization, respiratory assistance with oxygen (“moderate” Covid-19) or even intubation (“severe” Covid-19), often with long-term sequelae or a fatal outcome [3, 4]. Cancer is one of the established risk factors for severe Covid-19 [5], which may be related to the facts that (i) malignant disease mostly develops in aged individuals, often in the context of other comorbidities [6–8] (ii) cancer manifests preferentially in the context of failing immunosurveillance and mediates local and systemic immunosuppressive effects during tumor progression [9, 10]; and (iii) antineoplastic therapies, in particular combination chemotherapies, have debilitating, pro-inflammatory, immunosuppressive and senescence-accelerating side effects [11, 12].

Multiple studies have attempted to identify biomarkers that distinguish patients likely to develop mild *versus* severe Covid-19 [1, 2, 13]. Obviously, such biomarkers are linked to deficient anti-SARS-CoV-2 immune responses (such as severe lymphopenia or, more specifically, inefficient type-1 interferon responses due to inherent immune defects or the production of autoantibodies that neutralize type-1 interferons) [14–16] or excessive inflammatory responses (that often manifest at the levels of granulocytes, monocytes and their products) [17, 18]. The search of predictive biomarkers has been involving advanced technologies including high-dimensional cytometry [17], single-cell transcriptomics [19] and proteomics [20]. An additional unbiased strategy for defining circulating factors useful for risk stratification is mass spectrometric metabolomics, a technique that requires a minimum of initial sample preparation (snap freezing of serum or heparin serum and its storage at -80° C), is entirely automatable and yields accurate information on hundreds of known metabolites (i.e. a combination of chromatographic retention times and masses that allow for the *>bona fide* identification of the corresponding chemical compound) as well as thousands of unknown metabolites [21–32].

Here, we used mass spectrometric metabolomics to identify biomarkers of Covid-19 severity in cancer patients recovered at the Gustave Roussy Cancer Campus, which comprises the largest cancer-specific research hospital in Europe. We identified a series of metabolites that correlate with disease severity in cancer patients.

## RESULTS

### Study design and metadata

**Figure 1:**
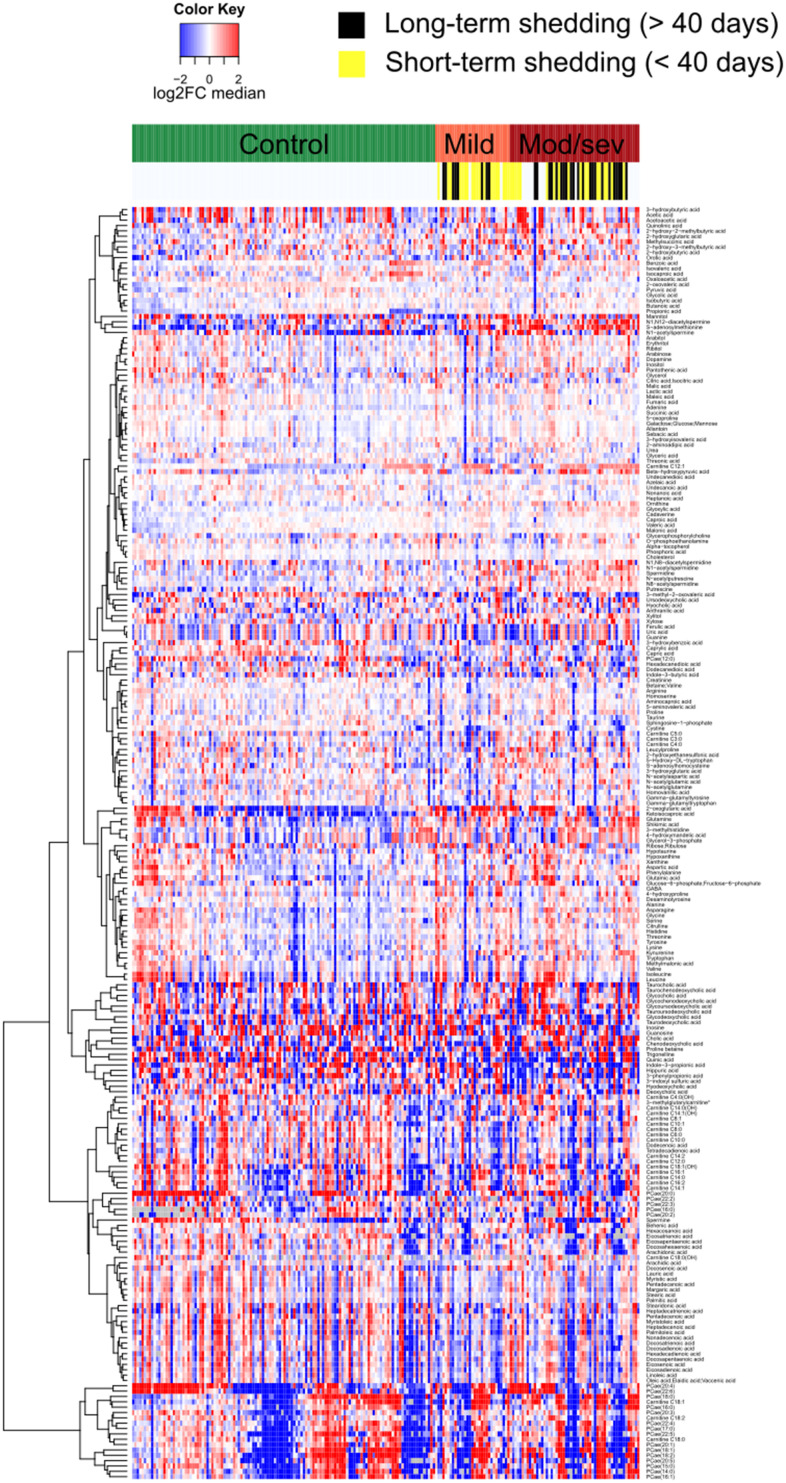
**Heatmap representing the serum metabolome of each individual cancer patient clustered by clinical severity of Covid-19.** Targeted metabolomic data on 211 serum samples from 204 patients were normalized areas of identified metabolites. Results are listed in Supplementary Table 1.

We determined the metabolome of serum samples from 204 cancer patients enrolled in the ONCOVID trial (https://clinicaltrials.gov/ct2/show/NCT04341207). Patients were divided into “controls” (no diagnosis of Covid-19, no signs of respiratory infection) and two categories of PCR-confirmed Covid-19 patients with “mild disease” (with ambulatory treatment) or “moderate/severe disease” (requiring hospitalization and respiratory assistance). Indeed, the number of patients with severe Covid-19 (requiring intubation and mechanical ventilation) was too low to be analyzed as a separate group. Importantly, the duration of viral shedding (short-term shedding: <40 days, long-term shedding: >40 days) was variable among Covid-19 infected cancer patients, yet tended to be shorter in mild than in moderate/severe cases (Figure 1) as already observed by Goubet et al. [33]. The clinical characteristics of both cohorts are summarized in Table 1. Serum samples from both cohorts were subjected to mass spectrometric metabolomics, yielding high-quality information on 239 identified metabolites (Figure 1 and Supplementary Table 1). Moreover, the cohort yielded 4276 non-identified mass spectrometric peaks (Supplementary Figure 1 and Supplementary Table 2).

**Table 1:** Clinical characteristics of SARS-CoV-2 patients affected by cancer.

**Cancer patient characteristics**		**All N=204**	**Control n=128**	**COVID-19**		***p***
				**Mild n=37**	**Moderate or severe** **n=39**	
Age (year)	Median (range)	61 (19-89)	60 (19-83)	65 (19-89)	64 (33-83)	*-*
Gender – no (%)	Male	84 (41)	54 (42)	12 (32)	18 (46)	*0.44*
	Female	120 (59)	74 (58)	25 (68)	21 (54)	
Number of comorbidities – no (%)	0	104 (51)	71 (55)	16 (43)	17 (44)	*0.42*
	1	51 (25)	27 (21)	14 (38)	10 (26)	
	2	30 (15)	18 (14)	4 (11)	8 (21)	
	>3	19 (9)	12 (9)	3 (8)	4 (10)	
Comorbid conditions – no (%)	COPD	15 (7)	13 (10)	1 (3)	1 (3)	*0.51*
	Obesity (BMI ≥ 30)	18 (9)	11 (9)	2 (5)	5 (13)	
	Hypertension	66 (32)	37 (29)	15 (41)	14 (36)	
	Congestive heart failure	7 (3)	4 (3)	1 (3)	2 (5)	
	Diabetes mellitus	22 (11)	11 (9)	5 (14)	6 (15)	
Type of malignancy – no (%)	Solid tumors	180 (88)	121 (95)	31 (84)	28 (72)	*0.0004*
	Hematological malignancies	24 (12)	7 (5)	6 (16)	11 (28)	
Cancer spread - no (%)	Localized	89 (44)	68 (53)	11 (30)	10 (26)	*0.002*
	Locally advanced	25 (12)	11 (9)	9 (24)	5 (13)	
	Metastatic	90 (44)	49 (38)	17 (46)	24 (61)	
ECOG performance status – no (%)	0	108 (53)	81 (63)	21 (57)	6 (15)	*<0.0001*
	1	52 (25)	33 (26)	6 (16)	13 (33)	
	2 or more	44 (22)	14 (11)	10 (27)	20 (51)	
Death – no (%)	Yes	45 (22)	27 (21)	5 (14)	13 (33)	*0.10*

SVS, Short Viral Shedding; LVS, Long Viral Shedding; no, number; COPD, Chronic Obstructive Pulmonary Disease; Ct, Cycle threshold. Statistic tests, Mann-Whitney or Chi-Square.

### Depletion of two bacterial propionate derivatives in moderate/severe Covid-19

**Figure 2:**
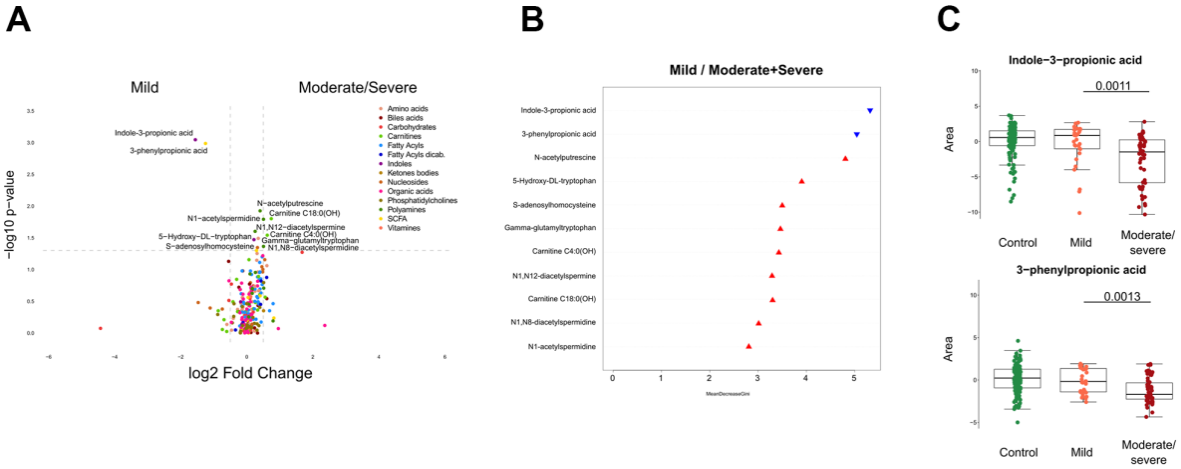
**Identification of metabolites discriminating cancer patients by clinical severity of SARS-CoV-2.** (**A**) Volcano plot comparing mild with moderate/severe Covid-19 patients, classified by chemical classes. X-axis: log^2^ fold change of metabolites; Y-axis: fold change of –log^10^ P value determined by the Mann–Whitney test. (**B**) Random forest classification model based on metabolites altered (p < 0.05) between mild and moderate/severe Covid-19 cases. The downregulated and upregulated metabolites in mild compared to moderate/severe patients are marked in blue and red, respectively. (**C**) Indole-3-propionic acid and 3-phenylproprionic levels in patients. All boxes indicate the interquartile range Q1 to Q3 with Q2 (median levels) in the center. The range of outliers is depicted by whiskers. The black bar of the figure indicates the p-value.

To identify metabolites the abundance of which increases or decreases with disease severity, we generated volcano plots that pinpoint variations in the metabolite concentration by at least 20% (up or down) with a p-value ≤0.05 (Figure 2A). Eleven metabolites fulfilled these criteria (Figure 2A) and were then subjected to random forest classification to identify which among them have the best predictive values (Figure 2B). Of note, two chemically related compounds, indole-3-proprionate and 3-phenylproprionate were reduced in cancer patients with moderate/severe Covid-19 compared to cancer patients with mild disease. A prior study has shown that critically ill patient with pneumonia have low serum levels of 3-phenylproprionate, a bacterial product that may be depleted as a result of antibiotic use [34]. Moreover, it is known that indole-3-proprionate is produced by Clostridium sporogenes in the human gastrointestinal tract [35, 36] and that low indole-3-proprionate serum levels reflect a diet poor in fibers [37] and a low microbiome diversity [38]. Thus, the depletion of indole-3-proprionate and 3-phenylproprionate (Figure 2C) that was found may indicate a state of intestinal dysbiosis.

### Identification of disease severity-associated acetylated polyamine derivatives

**Figure 3:**
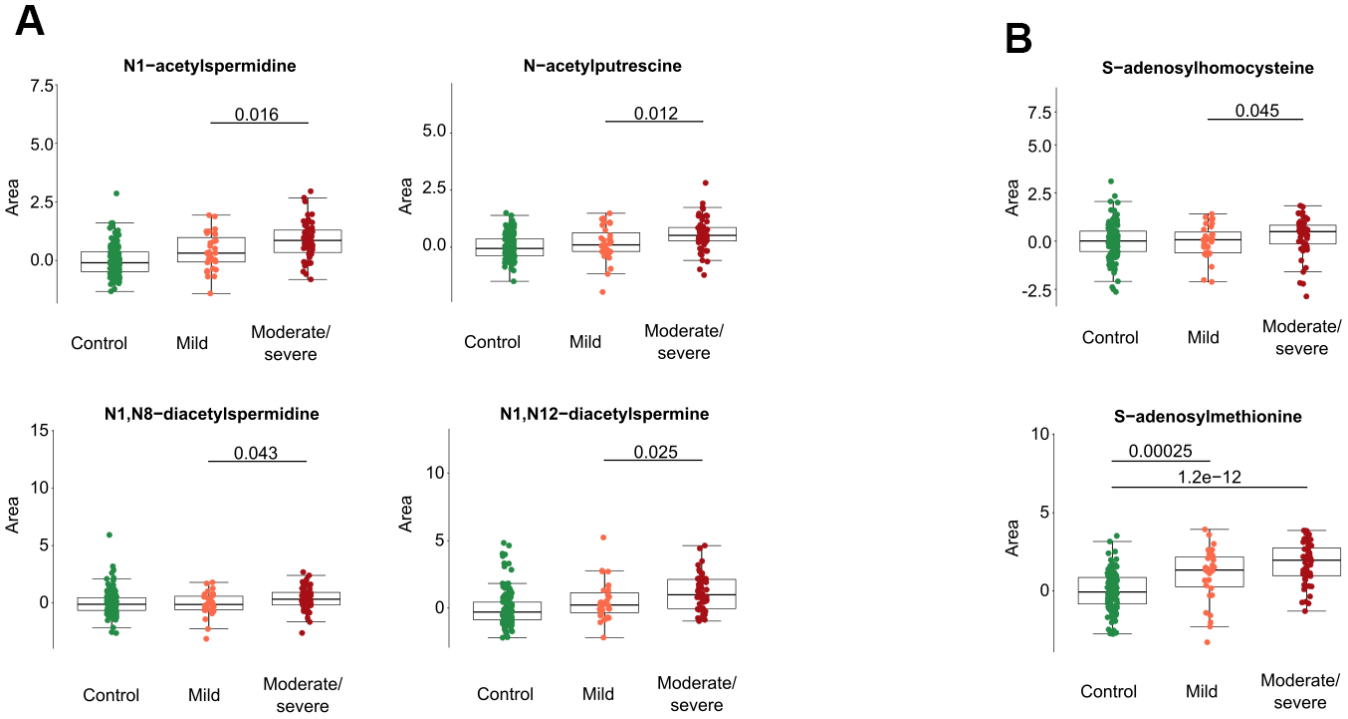
**Acetylated polyamine derivatives and associated metabolites in cancer patients with different levels of Covid-19 severity.** Acetylated polyamine derivatives were identified by targeted metabolomics data (**A**). S-adenosylhomocysteine and S-adenosylmethionine are shown (**B**). All data represent the normalized areas of mass spectrometric peaks and were analyzed by non-parametric unpaired Wilcoxon test (Mann–Whitney) for each two-group comparison. The black bar of the figure indicates the p-value.

**Figure 4:**
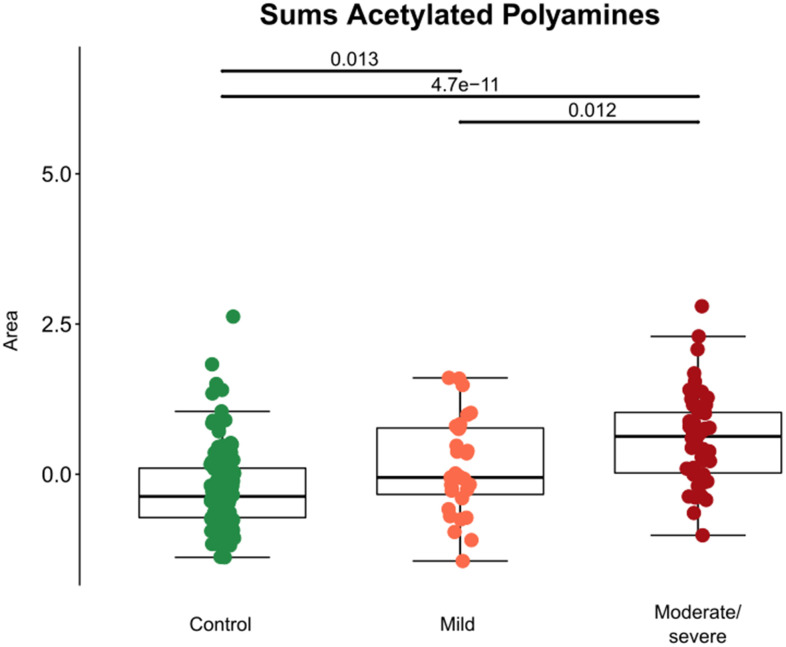
**Aggregate analyses of acetylated polyamine derivatives in cancer patients with different levels of Covid-19 severity.** For each patient, the sum of the normalized peak areas corresponding to N_1_-acetylputrescin, N_1_-acetylspermidine, N_1_,N_12_-diacetylspermine and N_1_,N_8_-diacetylspermidine were calculated and shown with black bars indicating p-values.

**Figure 5:**
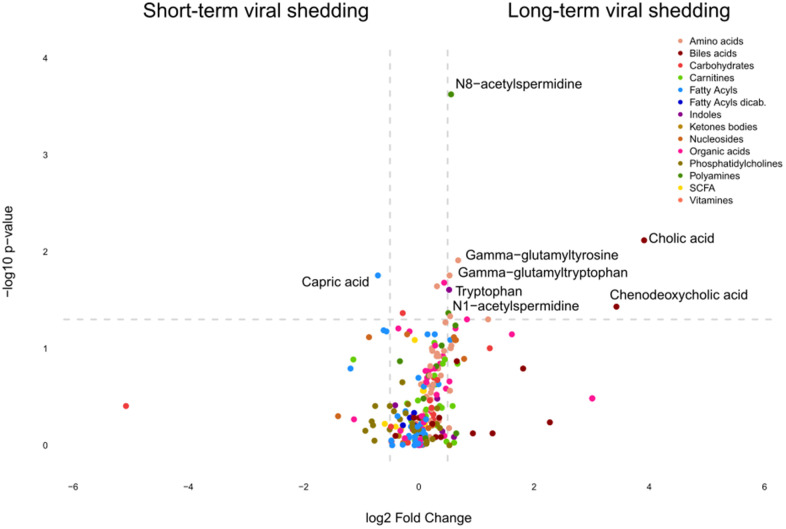
**Identification of metabolites discriminating cancer patients according to the duration of SARS-CoV-2 shedding.** Volcano plot comparing Covid-19 patients with short *versus* long viral shedding (determined by RT-PCT of nasopharyngeal PCRs, the threshold between short and long shedding being 40 days), classified by chemical classes. X-axis: log^2^ fold change of metabolites; Y-axis: fold change of –log^10^ P value determined by the Mann–Whitney test.

Several acetylated polyamine derivatives (N_1_-acetylputrescine, N_1_-acetylspermidine, N_1_,N_8_-diacetylspermidine and N_1_,N_12_-diacetylspermine) were overabundant in moderate/severe compared to mild Covid-19 (Figures 2A, 3A) when they were analyzed individually (Figure 3A). S-adenosylmethionine, a metabolite that is connected to polyamine synthesis [39], also correlated with Covid-19 severity (Figure 3B) echoing a prior report [40]. The random forest calculations based on all metabolites that changed significantly based on the volcano plot analyses (Figure 2B) yielded a classification model with an out-of-bag (OOB) error rate of 22.35% (Table 2). The boxplot representation illustrates that acetylated polyamine derivatives tend to be higher in Covid-19 infected patients than in controls and that they significantly increase with disease severity (Figure 3A, 3B). The ratio of N_1_-acetylspermidine over spermidine, as well as the ratio of N_1_,N_8_-diacetylspermine over spermidine, increased, but no such increase was found for the ratios of N_1_-acetylputrescine over putrescine and N_1_,N_12_-diacetylspermine over spermine (which was actually reduced) (Supplementary Figure 2). The sum of all acetylated polyamines (N_1_-acetylputrescine + N_1_-acetylspermidine + N_1_,N_8_-diacetylspermidine + N_1_,N_12_-diacetylspermine) yielded a lower discriminative p-value than each of them alone (Figure 4). Altogether, these results support the idea that acetylated polyamine derivatives correlate with Covid-19 severity. When the duration of PCR-detectable SARS-CoV-2 shedding was used to distinguish short-term carriers (<40 days) from long-term-carriers (>40 days), the serum levels of N_1_-acetylspermidine and N_8_-acetylspermidine were found to be slightly but significantly increased in long-term carriers (Figure 5). Thus, failure to eliminate SARS-CoV-2 is associated with an increase of selected acetylpolyamines.

**Table 2:** Error rate estimates for the Random Forest SARS-CoV-2.

	**Prediction: OOB estimate of error rate: 22.35%**			
		***Predicted label***		
*Actual label*	**Label**	**Mild**	**Moderate/severe**	**Class error**
	**Mild**	16	15	48.39%
	**Moderate/severe**	4	50	7.41%

### Correlations between circulating metabolites and cytokines

**Figure 6:**
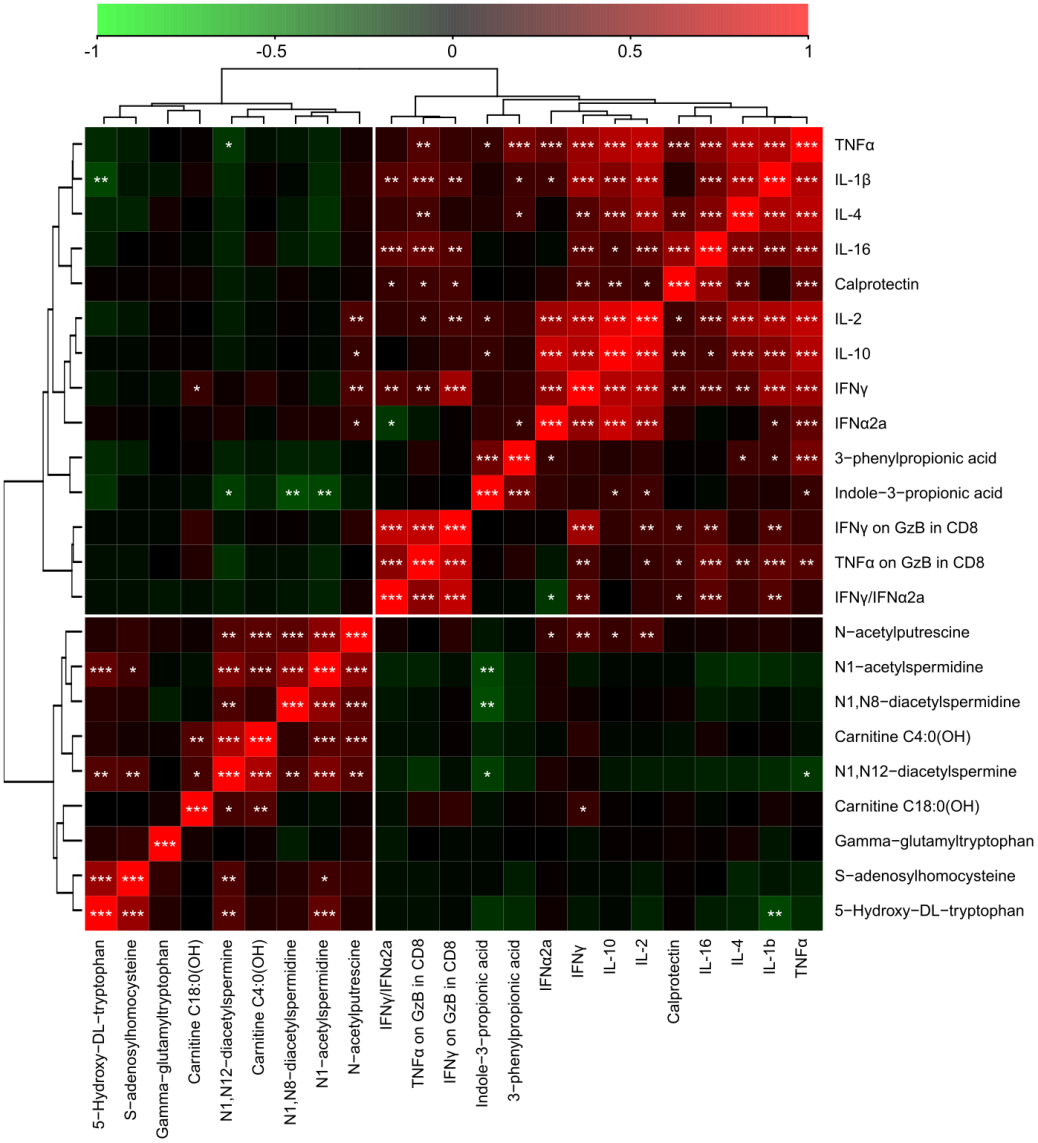
**Integration of metabolic with inflammatory markers in serum samples from cancer patients with different levels of Covid-19 severity.** The correlation heatmap of data was generated by means of Pearson’s method, and clustered using the ward. D2 method. The red color indicates positive correlations with a FDR<0.05, and the green color marks negative correlations with FDR<0.05. Black indicates non-significant (FDR>0.05) associations. *p < 0.05, **p < 0.01, ***p < 0.001. TNFα: tumor necrosis factor alpha. GzB: Granzyme B. TNFα on GzB in CD8 is the ratio between soluble TNFα and GzB in CD8+ T cells (by flow cytometry) and IFNγ on GzB in CD8 is the ratio between soluble IFNγ GzB in CD8+ T cells (by flow cytometry).

**Figure 7:**
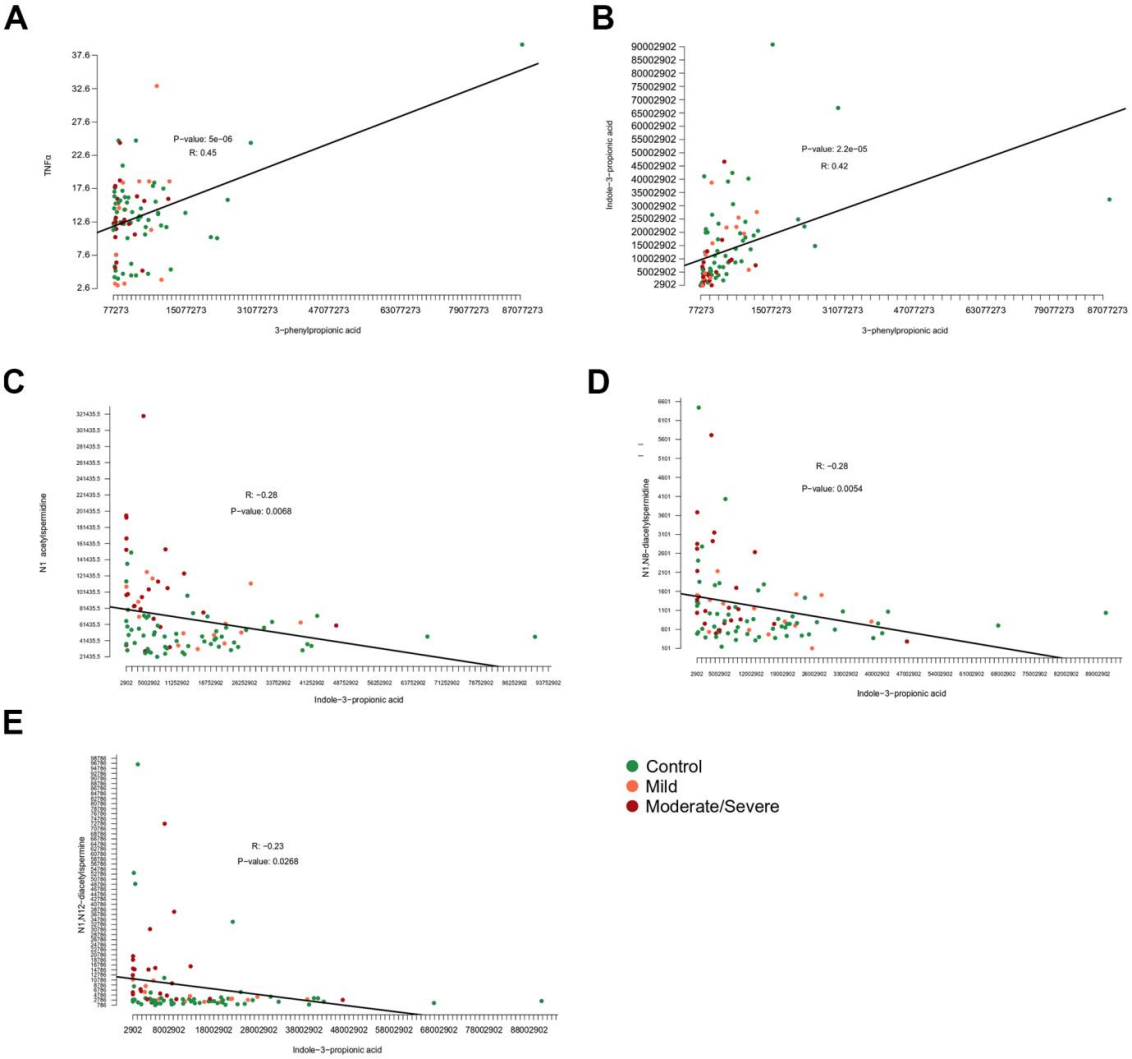
**Correlation among metabolites and inflammatory markers.** Correlations of (**A**) 3−phenylpropionic acid and tumor necrosis factor alpha (TNFα), (**B**) 3−phenylpropionic acid and indole-3-phenylpropionic acid, (**C**) indol-3-propionic acid and N_1_-acetylspermidine or (**D**) N_1_,N_8_-diacetylspermidine or (**E**) N_1_,N_12_-diacetylspermine.

In the final step of data analysis, we established a correlation matrix to visualize positive or negative associations among significantly altered metabolites and cytokines that were measured in the serum from patients enrolled (Figure 6). Non-hierarchical clustering revealed positive associations among several acetylpolyamines, SAM, as well 5-hydroxy-tryptophan and 2 saturated acyl carnitines (butyryl-L-carnitine, arachidyl-L-carnitine with 4 and 20 carbon atoms in the acyl chain, respectively). Among the acetylated polyamines, N_1_-acetylputrescine correlated with several cytokines in particular, the interferons (IFN) IFNα2a and IFNγ, as well as the interleukins (IL) IL-2 and IL-10, but no such correlation was found for any of the other acetylpolyamines (N_1_-acetylspermidine, N_1_,N_8_-diacetylspermidine and N_1_,N_12_-diacetylspermine). All immune and inflammation-related parameters clustered together, including a positive association between indole-3-propionate and 3-phenylpropionate (Figure 6 and Supplementary Figures 3–6). In particular, the association between 3-phenylproprionate and circulating tumor necrosis factor-α (TNFα) was highly significant (Figure 7A). Moreover, this bacterial metabolite exhibited a positive correlation with indole−3−propionate (Figure 7B), which in turn exhibited a negative correlation with N_1_-acetylspermidine, N_1_,N_8_-diacetylspermidine and N_1_,N_12_-diacetylspermine (Figure 7C–7E). This anticorrelation supports the general conclusions of this paper.

## DISCUSSION

Here, we present an unbiased metabolomics-based approach to identify circulating metabolites that are elevated in severe Covid-19, in the context of cancer. Our findings indicate that some bacterial metabolites are depleted in severe Covid-19, suggesting the presence of intestinal dysbiosis. Whether this dysbiosis results from severe SARS-CoV-2 infection, reflects a preexisting condition or results from antibiotic treatments remains to be determined.

Prior studies performed on general (non-cancer-specific) patient cohort have revealed a disease severity-associated increase in the tryptophan metabolite kynurenine [24, 28, 30, 41], which is well known for its immunosuppressive properties. Indeed, addition of epacadostat, an inhibitor of the kynurenine-generating enzyme indole 2,3-dioxygenase 1 (IDO1) has been shown to suppress the SARS-CoV-2-induced proinflammatory cytokine release *ex vivo* [31].

**Figure 8:**
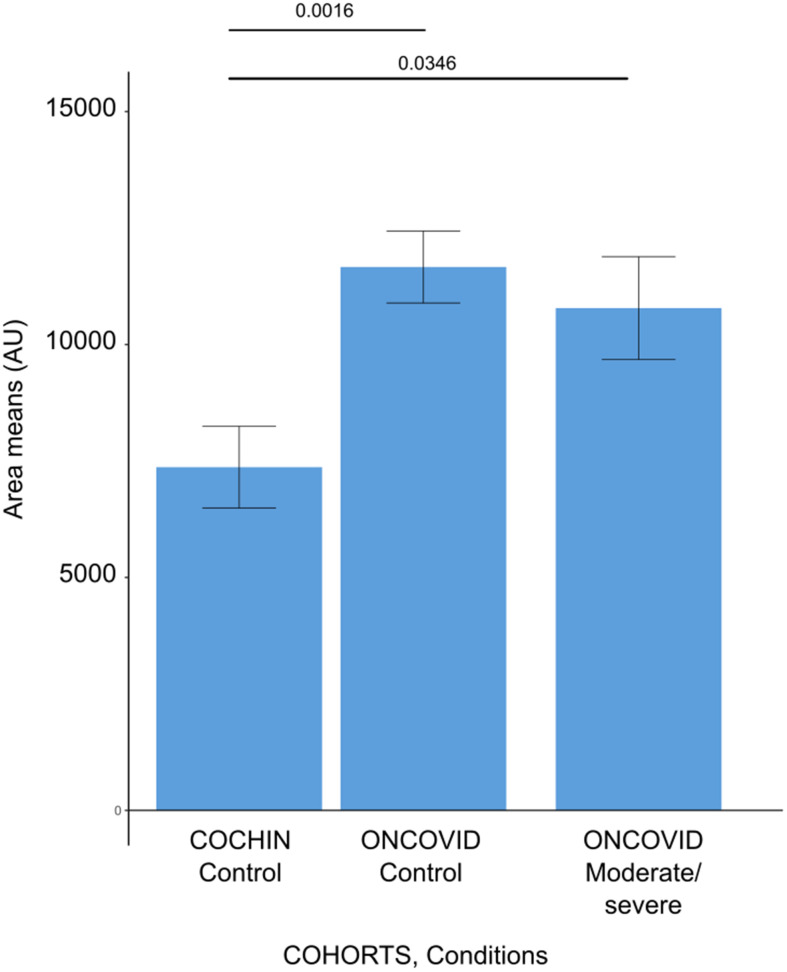
**Anthranilic acid levels in different patient cohorts.** Barplots demonstrating the anthranilic acid area levels for each subject issued from the cancer-free and Covid-19-free control cohort (COCHIN Control), previously described by Danlos et al., [45], and our dataset of cancer patients without Covid-19 (ONCOVID controls) and the cancer patients with moderate or severe Covid-19 (ONCOVID Moderate/Severe). Error bars show standard errors of the mean.

Interestingly, high levels of kynurenine metabolites were identified in the murine serum metabolome after infection by H1N1 influenza virus infection, suggesting that distinct viruses may affect this inflammation-relevant metabolic pathway [42]. In our work, a general (non-cancer-specific) cohort, we found that another immunosuppressive tryptophan derivative, anthranilic acid [43, 44], had a poor prognostic value, correlating with the maintenance of high interleukin-10 and -18 levels [45]. In sharp contrast, we did not find anthranilic acid to be increased in Covid-19 patients with cancer, most likely because the levels of this immunosuppressive metabolite are already increased at baseline, in Covid-19-free cancer patients as compared to cancer-free control individuals (Figure 8). This suggests that malignant disease is associated with an augmentation of circulating anthranilic acid concentrations. Indeed, some cancers such as HER2-positive and triple-negative mammary carcinomas overexpress the enzymes kynurenine 3-monooxygenase (KMO) and kynureninase (KYNU), causing increased production of anthranilic acid [46]. Moreover, poor prognosis prostate cancer is associated with elevated circulating anthranilic acid levels [47]. However, at this point the causes of the elevation of anthranilic acid across different cancer types remain unclear.

The most important finding of this study concerns the Covid-19 severity-associated surge in acetylated polyamines found in cancer patients. Increased acetylated polyamine levels, in particular mono- and diacetyl spermidine and spermine derivatives (such as N_1_-acetylspermidine, N_1_,N_8_-diacetylspermidine and N_1_,N_12_-diacetylspermine), have been found in cancer-free cohorts of Covid-19 patients to be associated with disease severity [40, 45]. Hence, this alteration appears to be a general feature of moderate or severe Covid-19 infection requiring hospitalization, irrespective of the presence or absence of neoplasia.

However, the functional implications of these findings are elusive. Spermidine inhibits SARS-CoV-2 replication *in vitro* [48]. Spermidine supplementation is known to exert immunostimulatory effects and to improve the effects of anticancer chemotherapy and immunochemotherapy [39, 49–52]. However, acetylated polyamine derivatives have not been investigated with respect to their potential immunomodulatory effects.

It is important to note that acetylated polyamine derivatives have previously been associated with the risk of developing hepatocellular carcinoma [53], poor prognosis triple-negative breast cancer [54], non-small cell lung cancer [55, 56], colorectal cancer [57], pancreas carcinoma [58], lethal cardiovascular disease [59, 60], Parkinson disease [61] and an elderly-type gut microbiota [62]. This contrasts with the observations that nutritional uptake of spermidine (and spermine but not putrescine) is epidemiologically linked to a decrease in the risk of lethal cancer, cardiovascular disease and cognitive decline [39, 63] and that experimental spermidine supplementation has wide oncopreventive, cardioprotective and neuroprotective effects in preclinical models [64–68].

It is possible that the elevated levels of mono- and di-acetyl spermidine reflect a higher spermidine catabolism, reducing the bioavailability of endogenous spermidine. Indeed, intracellular spermidine and spermine are acetylated by spermidine/spermine N_1_-acetyltransferase-1 (SAT1) and the resulting acetylated products can be either extruded from the cell or oxidized to putrescine [39, 63]. Thus, transgenic overexpression of SAT1 or its pharmacological activation depletes spermidine and spermine but increases the levels of mono- or diacetylated spermidine and spermine while limiting cellular proliferation and favoring the induction of apoptosis [69, 70]. Inhibition of SAT1 reduces acetyl-coenzyme A consumption in white adipose tissue or the liver, thus enhancing lipogenesis, while its activation stimulates beige adipocyte biogenesis as well as the expression of pro-inflammatory genes [71–73]. Transgene-enforced overexpression of SAT1 accelerates aging in mice [74] as it enhances carcinogenesis [75, 76], while its knockout protects against liver and kidney ischemia-reperfusion damage [77], CCL4-induced acute liver injury [78], and endotoxin- or cisplatin-induced acute kidney injury [79, 80]. Enhanced expression of SAT1 is a biomarker of kidney ischemia-reperfusion damage [81], poor prognosis prostate cancer [82], and radioresistance in brain tumors [83]. Amantadine, a clinically approved antiviral drug, is a substrate for SAT1 and hence can be used to indirectly measure SAT1 activity by assessing the quantity of urinary acetylamantadine. Using this test, patients with breast or lung cancer exhibit an enhanced SAT1 activity [84]. Of note, SAT1 is upregulated by interferons [85], perhaps explaining the shift in polyamine metabolism associated with severe Covid-19. On theoretical grounds, the depletion of intracellular spermidine resulting from SAT1 activation may favor SARS-CoV-2 replication [48] and enfeeble the antiviral immune response due to the depletion of bioavailable spermidine [49, 51, 52, 83, 86]. However, the effects of polyamine catabolism on infections by respiratory viruses have not yet been studied in suitable animal models.

In conclusion, it appears that the severity of Covid-19 affecting cancer cells is strongly related to the acetylation of polyamines, in particular that of spermidine and spermine. It will be important to understand the mechanisms as well as the functional consequences of this over-acetylation detectable in cancer patients.

## MATERIALS AND METHODS

### Standards and reagents

Acetonitrile (Sigma Aldrich)

Isopropanol (Sigma Aldrich)

Methanol (Sigma Aldrich)

Chloroform (Sigma Aldrich)

Acetic acid (Sigma Aldrich)

Formic acid (Sigma Aldrich)

Methoxyamine hydrochloride (Sigma Aldrich)

MSTFA - N-Methyl-N-(trimethylsilyl) trifluoroacetamide (Sigma Aldrich)

Pyridine (Sigma Aldrich)

3 nitrophenylhydrazine (Sigma Aldrich)

*N*-(3-Dimethylaminopropyl)-*N*′-ethylcarbodiimide hydrochloride (EDC) (Sigma Aldrich)

Sulfosalicylic acid (Sigma Aldrich)

### Study ONCOVID design and participants

Gustave Roussy Cancer Center mentored the “ONCOVID” trial and collaborated with the academic authors. All patients provided written informed consent. This clinical trial was conducted in accordance with the principles of the Declaration of Helsinki. Protocol approval was obtained from an independent ethics committee (ethics protocol number EudraCT No: 2020-001250-21). The protocol is available with the full text of this article at https://clinicaltrials.gov/ct2/show/NCT04341207.

Blood samples were drawn from patients enrolled in ONCOVID at Gustave Roussy Cancer Campus (Villejuif, France). Whole human peripheral blood was collected into sterile vacutainer tubes. Serum were collected after centrifugation at 600 × g for 10 min at room temperature and transferred to −80° C freezer to await analysis. Serum samples were used to perform metabolomic approaches and monitor the concentrations of soluble factors. Fixed whole blood were used for spectral flow cytometry. For details, please referred from previously described by Goubet et al. [33].

### Evaluation of SARS-CoV-2 RNA shedding

The duration of viral shedding was defined as the number of days from the first positive to the first negative RT-qPCR, after longitudinal monitoring. In order to prevent an overvaluation of this duration, we considered in this analysis only patients with an interval below 40 days between the last positive RT-qPCR and the first negative RT-qPCR. Six patients had one negative RT-qPCR followed by positive RT-qPCR. We extend the duration to the second negative RT-qPCR for 3 patients with a cycle threshold below 35 for the gene coding replication-transcription complex and within 6 days after the first negative result [33].

### Sample preparation serum (dry tubes)

A volume of 50 μL of serum were mixed with 500 μL a cold solvent mixture with ISTD (MeOH/Water, 9/1, -20° C), into 1.5 mL microtubes, vortexed and centrifuged (10 min at 15000 g, 4° C) to obtain protein precipitation. Then upper phase of supernatant was split in three parts: 150 μL were used for GC-MS experiment in injection vial, 40 μL were used for the SCFA (Short Chain Fatty Acids) UHPLC-MS method, and 150 μL were used for others UHPLC-MS experimentations as described [45]. Extracted biological samples were pooled in quality controls samples, used for data correction during the data treatment.

### Widely-targeted analysis of intracellular metabolites gas chromatography (GC) coupled to a triple quadrupole (QQQ) mass spectrometer

GC-MS/MS method was performed on a 7890B gas chromatography (Agilent Technologies, Waldbronn, Germany) coupled to a triple quadrupole 7000C (Agilent Technologies, Waldbronn, Germany) equipped with a High sensitivity electronic impact source (EI) operating in positive mode [45]. The scan mode used was the MRM for biological samples. Peak detection and integration of analytes were performed using the Agilent Mass Hunter quantitative software (B.07.01), exported as tables and processed with R software (version 4.0.3) and the GRMeta package (Github/kroemerlab).

### Targeted analysis of bile acids by ion pairing high performance liquid chromatography (HPLC) coupled to a QTRAP 6500+ mass spectrometer

Targeted analysis was performed on a RRLC 1260 system (Agilent Technologies, Waldbronn, Germany) coupled to a QTRAP 6500+ (Sciex) equipped with an electrospray source operating in negative mode. The source conditions were: ion spray source temperature at 450° C, curtain (CUR) gas pressure at 25 psi, gas 1 (GS1) pressure at 30 psi and gas 2 (GS2) pressure at 70 psi.

2.5 μL of sample were injected on a Column Poroshell 120 EC-C8 (100 mm x 2.1 mm particle size 2.7 μm) from Agilent technologies, protected by a guard column XDB-C18 (5 mm × 2.1 mm particle size 1.8 μm) and heated at 40° C in a Pelletier oven.

Gradient mobile phase consisted of water with 0.2% of formic acid (A) and acetonitrile/isopropanol (1/1; v/v) (B) freshly made. Flow rate was set to 0.3 mL/min, and gradient as follow: initial condition was 70% phase A and 30% phase B, maintained during 1.5 min. Molecules were then eluted using a gradient from 30% to 60% phase B over 9 min. Column was washed using 98% mobile phase B for 2 minutes and equilibrated using 30% mobile phase B for 2 min. After each injection, needle was washed twice with isopropanol and thrice with water. The autosampler was kept at 4° C.

Collision gas was nitrogen. Scan mode used was the MRM for biological samples. Peak detection and integration of the analytes were performed using the Sciex MultiQuant quantitative software (Version 3.0.3), exported as tables and processed with R software (version 4.0.3) and the GRMeta package (Github/kroemerlab).

### Targeted analysis of polyamines by ion pairing ultra-high performance liquid chromatography (UHPLC) coupled to a triple quadrupole (QQQ) mass spectrometer

Targeted analysis was performed on a UHPLC 1290 system (Agilent Technologies, Waldbronn, Germany) coupled to a Triple Quadrupole 6470 (Agilent Technologies) equipped with an electrospray source operating in positive mode. The gas temperature was set to 350° C with a gas flow of 12 l/min. The capillary voltage was set to 2.5 kV.

10 μL of sample were injected on a Column Kinetex C18 (150 mm x 2.1 mm particle size 2.6 μm) from Phenomenex, protected by a guard column C18 (5 mm × 2.1 mm) and heated at 40° C in a Pelletier oven.

The gradient mobile phase consisted of water with 0.1 % of Heptafluorobutyric acid (HFBA, Sigma-Aldrich) (A) and acetonitrile with 0.1 % of HFBA (B) freshly made. The flow rate was set to 0.2 ml/min, and gradient as follow: initial condition was 95% phase A and 5% phase B. Molecules were then eluted using a gradient from 5% to 40% phase B over 10 min. The column was washed using 90% mobile phase B for 2.5 minutes and equilibrated using 5% mobile phase B for 4 min. The autosampler was kept at 4° C.

The collision gas was nitrogen. The scan mode used was the MRM for biological samples. Peak detection and integration of analytes were performed using the Agilent Mass Hunter quantitative software (B.07.01), exported as tables and processed with R software (version 4.0.3) and the GRMeta package (Github/kroemerlab).

### Targeted analysis of Short Chain Fatty Acid by ultra-high performance liquid chromatography (UHPLC) coupled to a Triple Quadrupole (QQQ) mass spectrometer

Targeted analysis was performed on a UHPLC 1290 system (Agilent Technologies, Waldbronn, Germany) coupled to a Triple Quadrupole 6470 (Agilent Technologies) equipped with an electrospray source operating in negative mode. The gas temperature was set to 300° C with a gas flow of 12 l/min. The capillary voltage was set to 5000 kV.

10 μL of sample were injected on a Column Zorbax Eclipse XBD C18 (100 mm x 2.1 mm particle size 1.8 μm) from Agilent technologies, protected by a guard column XDB-C18 (5 mm × 2.1 mm particle size 1.8 μm) and heated at 50° C by a Pelletier oven.

Gradient mobile phase consisted of water with 0.01% of formic acid (A) and acetonitrile with 0.01% of formic acid (B). Flow rate was set to 0.4 mL/min, and gradient as follow: initial condition was 80% phase A and 20% phase B, maintained during 6 min. Molecules were then eluted using a gradient from 20% to 45% phase B over 7 min. Column was washed using 95% mobile phase B for 5 minutes and equilibrated using 20% mobile phase B for 4 min. Autosampler was kept at 4° C.

The collision gas was nitrogen. The scan mode used was the MRM for biological samples. Peak detection and integration of analytes were performed using the Agilent Mass Hunter quantitative software (B.07.01), exported as tables and processed with R software (version 4.0.3) and the GRMeta package (Github/kroemerlab).

### Pseudo-targeted analysis of metabolites by ultra-high performance liquid chromatography (UHPLC) coupled to a Q-Exactive mass spectrometer. Reversed phase acetonitrile method

The profiling experiment was performed with a Dionex Ultimate 3000 UHPLC system (Thermo Scientific) coupled to a Q-Exactive (Thermo Scientific) equipped with an electrospray source operating in both positive and negative mode and full scan mode from 100 to 1200 m/z. The Q-Exactive parameters were: sheath gas flow rate 55 au, auxiliary gas flow rate 15 au, spray voltage 3.3 kV, capillary temperature 300° C, S-Lens RF level 55 V. The mass spectrometer was calibrated with sodium acetate solution dedicated to low mass calibration.

10 μL of sample were injected on a SB-Aq column (100 mm × 2.1 mm particle size 1.8 μm) from Agilent Technologies, protected by a guard column XDB-C18 (5 mm × 2.1 mm particle size 1.8 μm) and heated at 40° C in a Pelletier oven. The gradient mobile phase consists of water with 0.2% of acetic acid (A) and acetonitrile (B). The flow rate was set to 0.3 mL/min. Initial condition is 98% phase A and 2% phase B. Molecules were then eluted using a gradient from 2% to 95% phase B in 22 min. The column was washed using 95% mobile phase B for 2 minutes and equilibrated using 2% mobile phase B for 4 min.

The autosampler was kept at 4° C.

Peak detection and integration were performed using the Thermo Xcalibur quantitative software (3.1.), exported as tables and processed with R software (version 4.0.3) and the GRMeta package (Github/kroemerlab).

In parallel, raw data files obtained by the pseudo-targeted analysis described above were used to perform unbiased profiling analyses using the Thermo Scientific™ Compound Discoverer™ small molecule identification software (version 3.1).

### Data analysis using Compound Discoverer™

After sample injection and data acquisition, raw data files were processed with Compound Discoverer software following a customized node-based workflow for identifying unknown compounds in metabolomics.

First, spectra selection and retention time alignment were performed, followed by removal of background noise and baseline correction. Next, the processing workflow found chromatographic peaks for unknown compounds (molecular weight, MW, x retention time, RT) extracting all relevant spectral and chromatographic information, to predict the elemental composition of the unknowns. The possible identity of the unknown compounds was then searched against selected MS databases, such as ChemSpider (from MS1 scans by using MW or predicted composition when available), mZcloud (MS/MS spectral library), built-in databases (custom, local libraries), and Metabolika or KEGG databases (metabolic pathway search). Annotations are assigned to the detected compounds, to rank putative database results. Finally, the software performed statistical analysis using a multivariate method approach, e. g. PCA (unsupervised), and data visualization, e.g. volcano plots.

Compounds found to be statistically different between groups, were checked, and all data was exported to R software (version 4.0.3) for data representation.

### Random forest calculation

Targeted data used for random forest calculation were areas corrected by means of quality controls, log^2^ transformed, and centered on the mean of the control samples (AreaCorrLog2Cen datasheet of the raw data, Supplementary Table 1).

We oriented this calculation on the severity levels of the patient, and focused on the differences of mild *versus* moderated + severe patients. Data were submitted to a Kruskal-Wallis test, and metabolites were selected according to fold changes and p-values. Mild *versus* moderate/severe. The two groups had a similar weight in the random forest calculation.

### Cytokine measurements

Serum samples and cytokines measurements were prepared according the Goubet et al., method [33]. In this study, only these cytokines considered are, IFN-γ, IL-10, IL-16, IL-1β, IL-2, IL-4, IL-6, TNF-α and Calprotectin and IFN-α2a.

## Supplementary Material

Supplementary Figures

Supplementary Table 1

Supplementary Table 2
