# Supplementary material for: Circulating acetylated polyamines correlate with Covid-19 severity in cancer patients
Source: Aging (Albany NY). 2021 Sep 13;13(17):20860–85. doi: 10.18632/aging.203525 (PMC8457559; doi:10.18632/aging.203525)
Supplement: Supplementary Figures [file aging-13-203525-s001.pdf]

SUPPLEMENTARY FIGURES

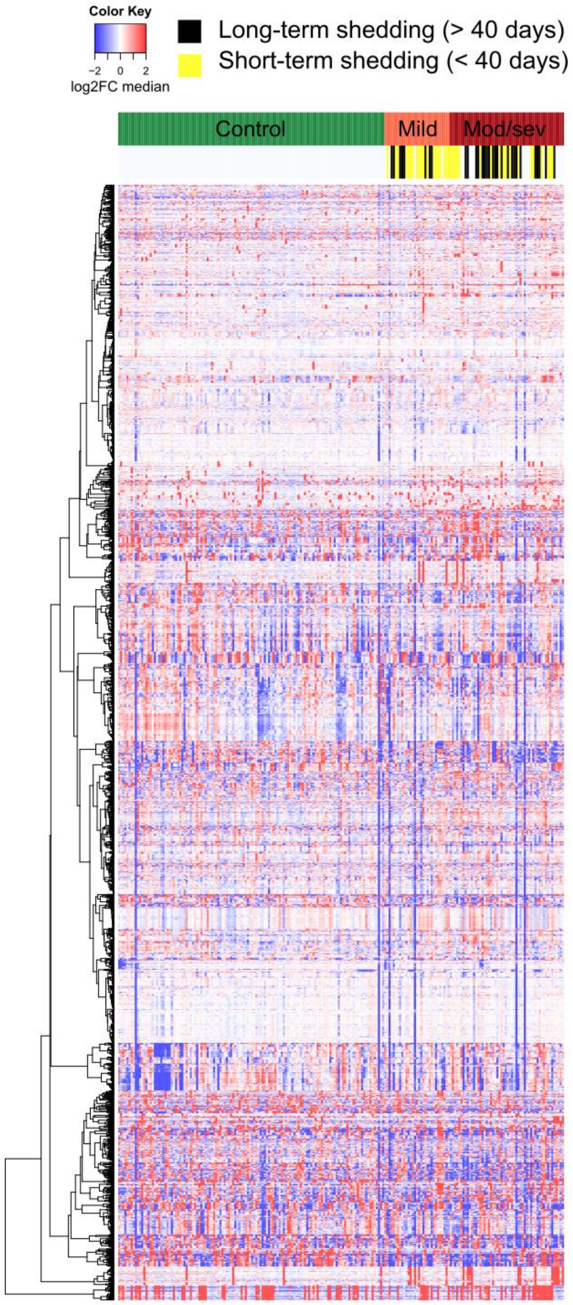

Supplementary Figure 1. Heatmap representing the serum metabolome of each individual cancer patients clustered by clinical severity of Covid-19. Untargeted metabolomic data on 211 serum samples from 204 patients were normalized area of non-identified mass spectrometric peaks. Results are listed in Supplementary Table 2.

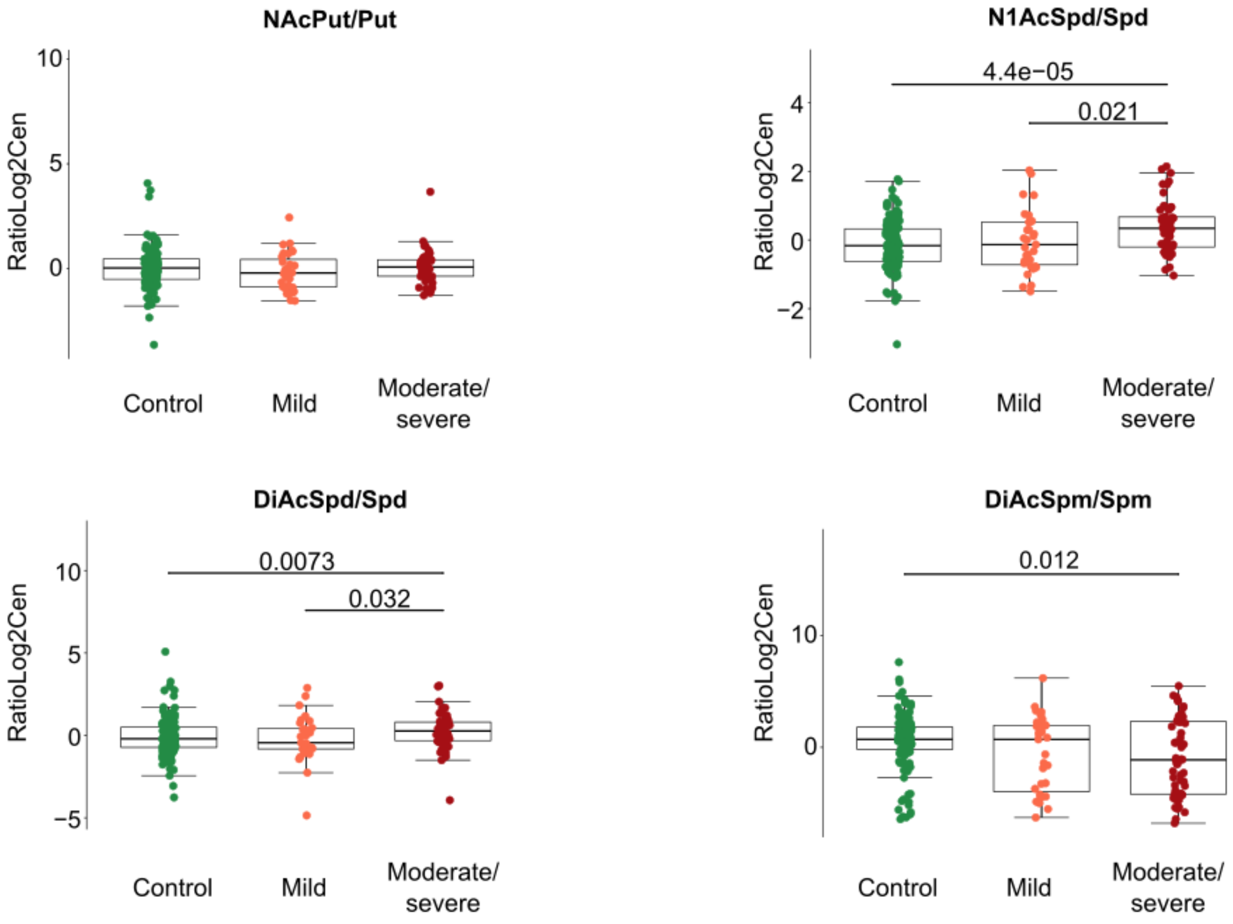

Supplementary Figure 2. Ratios of acetylated over non-acetylated polyamines in cancer patients with different levels of Covid-19 severity. The ratios were calculated as Log^2Cen of the areas of mass spectrometric peaks of N_1-acetylputrescin (NAcPut) over putrescine (Put), N_1-acetylspermidine (N_1AcSpd) or N_1,N_8-diacetylspermidine (DiAcSpd) over spermidine (Spd), or N_1,N_{12}-diacetylspermine (DiAcSpm) over spermine (Spm). Black bars indicate p-values.

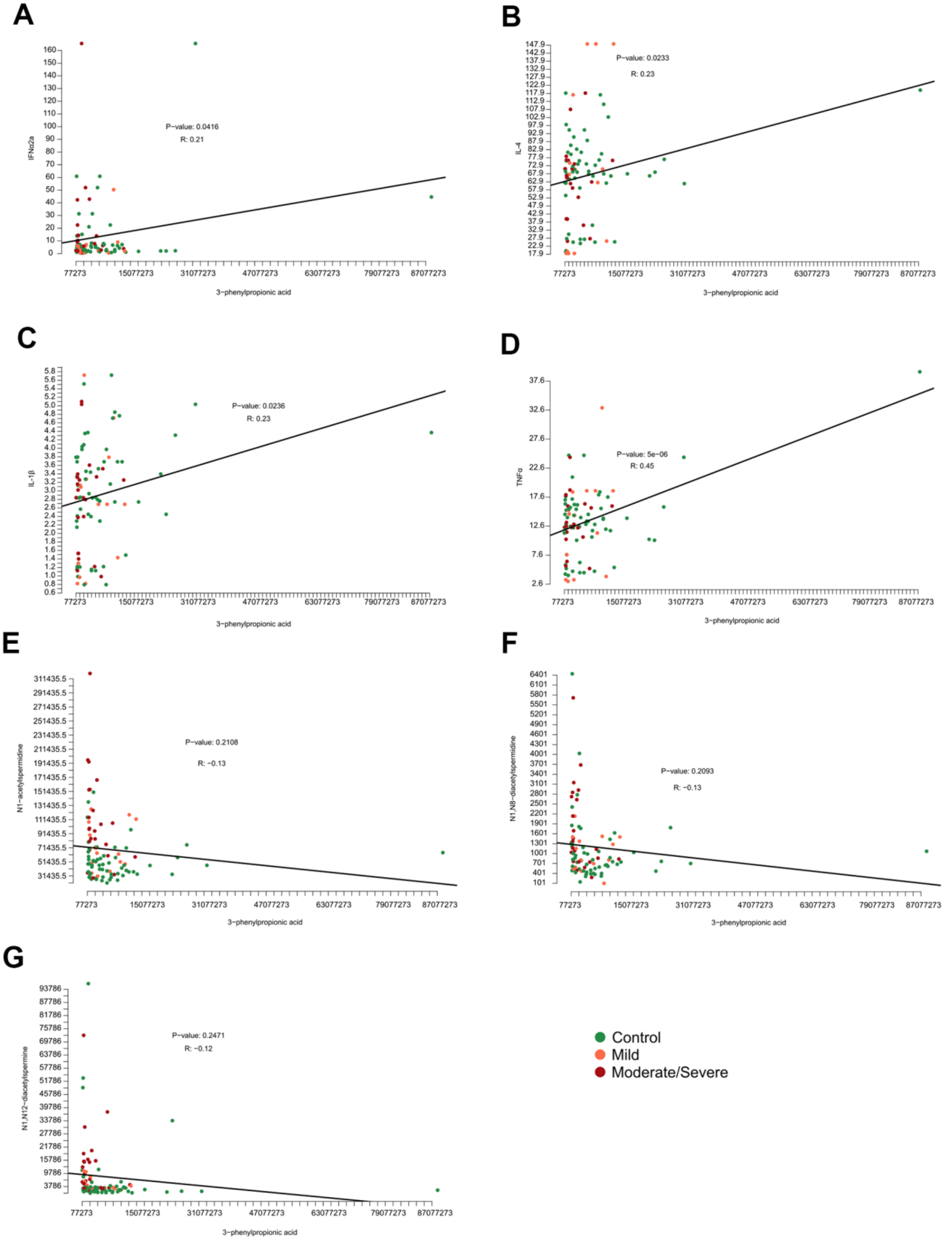

Supplementary Figure 3. Correlation of 3‒phenylpropionic acid with immune parameters. Levels of 3‒phenylpropionic acid was plotted against the concentrations of (**A**) interferon-α2a (IFNα2a), (**B**) interleukin-4 (IL-4), (**C**) Interleukin-1β (IL-1β), and (**D**) tumor necrosis factor alpha (TNFα), (**E**) N_1-acetylspermidine, (**F**) N_1,N_8-diacetylspermidine and (**G**) N_1,N_{12}-diacetylspermine.

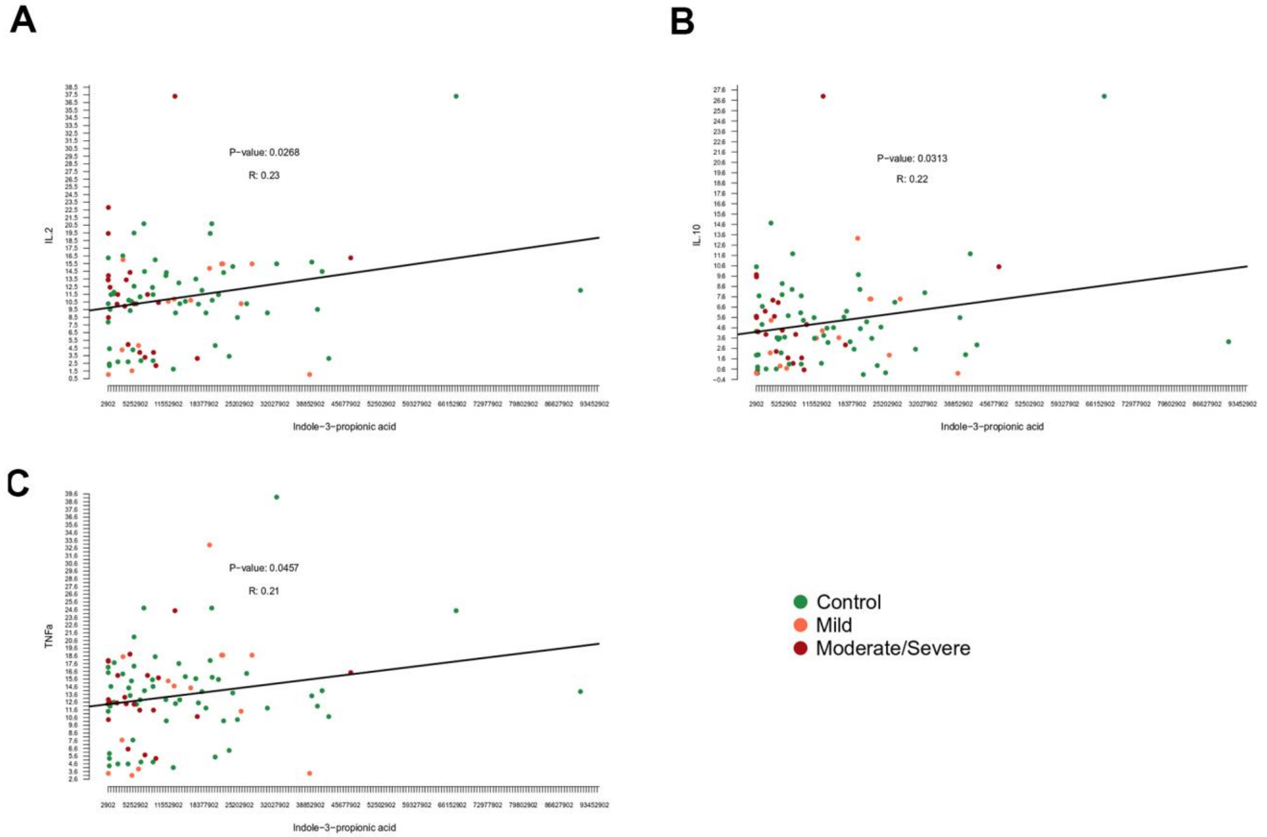

Supplementary Figure 4. Correlation of indole-3-propionic acid with immune parameters. Levels of indole-3-propionic acid was plotted against the concentrations of (**A**) interleukin-2 (IL-2), (**B**) interleukin-10 (IL-10), (**C**) tumor necrosis factor alpha (TNFα).

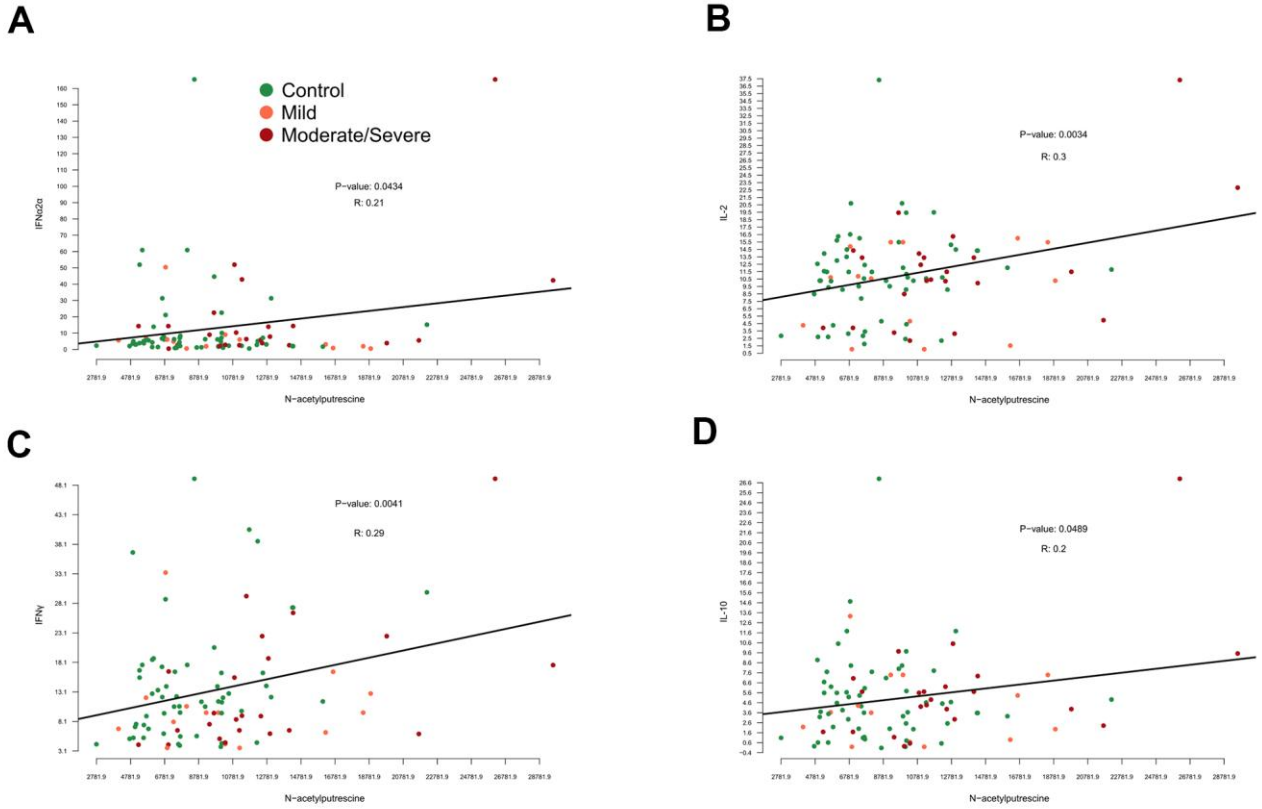

Supplementary Figure 5. Correlation of N‒acetylputrescine with immune parameters. Levels of N−acetylputrescine were plotted against the concentration of (**A**) interferon-α2a (IFNα2a), (**B**) interleukin-2 (IL-2), (**C**) interferon-γ (IFNγ) and (**D**) interleukin-10 (IL-10).

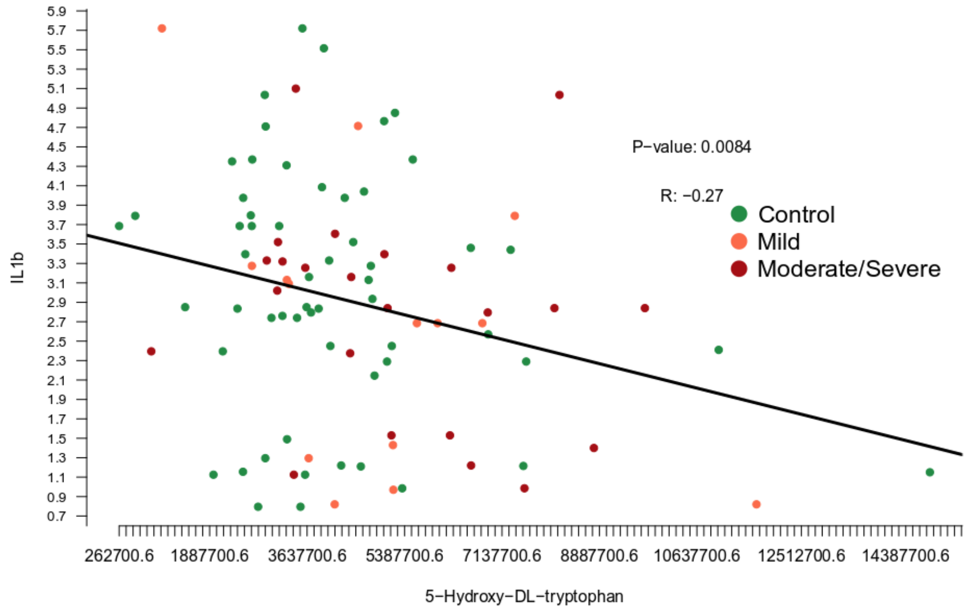

Supplementary Figure 6. Correlation of 5‒Hydroxy‒DL‒tryptophan with interleukin-1β (IL-1β) levels. Levels of 5‒Hydroxy‒DL‒tryptophan were plotted against the concentration of Interleukin-1β (IL-1β).
